# Mass and particle size distribution of household dust on children’s hands

**DOI:** 10.1038/s41370-025-00749-3

**Published:** 2025-02-10

**Authors:** Cristina Fayad-Martinez, Maribeth Gidley, Matthew A. Roca, Ryuichi Nitta, Ali Pourmand, Arash Sharifi, Foluke Adelabu, Jenna K. Honan, Olusola Olabisi Ogunseye, Paloma I. Beamer, Helena Solo-Gabriele, Alesia Ferguson

**Affiliations:** 1 Department of Chemical, Environmental, and Materials Engineering, University of Miami, Coral Gables, FL USA; 2 The Cooperative Institute For Marine and Atmospheric Studies, University of Miami, Miami, FL, USA; 3 Neptune Isotone Laboratory, Rosenstiel School of Marine, Atmospheric, and Earth Science, University of Miami, Miami, FL USA; 4 Research and Development Department, Isobar Science, Miami, FL USA; 5 Department of Built Environment, North Carolina Agricultural and Technical State University, Greensboro, NC USA; 6 Department of Community, Environment and Policy, Mel and Enid Zuckerman College of Public Health, University of Arizona, Tucson, AZ USA

**Keywords:** Dust loading, Children, Hands, Ingestion, Exposure, Household contaminants

## Abstract

**Background:**

Children are vulnerable to household dust exposure; however, to date, a handful of studies simultaneously report both the mass and particle size of household dust found on children’s hands after natural indoor play activities.

**Objective:**

Evaluate a new approach to measure dust loading and characterize particle size on a child’s hands using a Coulter Counter.

**Methods:**

The volume of particles rinsed off children’s hands was measured through counting and sizing particles (using a Coulter Counter), followed by multiplying the particle volume by the density of dust collected from the home. This mass was then normalized per total hand surface area to obtain dust loading on children’s hands. Results were compared by region (North Carolina, Florida, Arizona), age groups (6 months to 6 years), and social demographics (gender, race, ethnicity) for 101 children.

**Results:**

The estimated median density for household dust was 1.54 g/cm^3^, with an average of 1.58 g/cm^3^ (SD = 0.43). The overall median dust loading on children’s hands was 11.13 μg/cm^2^ (per total hand surface area), with a range of 0.004–167.6 μg/cm^2^. No statistical difference was observed by region, age, nor social demographics (p > 0.05). The majority of particles (90%) from children’s hand rinses had a diameter (D_90,v_) <35 μm; however, these small particles represent a fraction of the total mass. This new approach succeeded at obtaining dust loadings and particle size simultaneously from the same sample, in contrast to current methods that would have required multiple methods and sample types.

**Impact Statement:**

Children are vulnerable to household dust due to their play behavior; however, to date, limited measurements are available for the mass and particle size of dust on children’s hands after natural indoor play activities. We propose a new approach to facilitate dust loading measurements, while also obtaining the particle size of dust, through the usage of a Coulter Counter. Results showed that 90% of particles were <35 μm, which is four times smaller than the current guidelines threshold (150 μm) for risk assessments that utilize estimates for particles found on hands.

## Introduction

Household dust is a heterogeneous mixture which includes but is not limited to lint, skin particles, organic fibers, food debris, and soil from the outdoors. Within the indoor environment, it is also a significant repository of various harmful chemicals, including lead [1–11], pesticides [12], flame retardants [13–15], and emerging contaminants like per- and polyfluoroalkyl substances (PFAS) [16–19]. Exposure to household contaminants occurs through three primary routes: dermal contact [20], non-dietary ingestion (hands/objects in mouth) [21, 22], and inhalation [23], where the dust loading on hands is a critical factor for exposure estimates [8, 14]. With household dust having a natural tendency to adhere or deposit onto surfaces including skin, children are specifically at risk of exposure to household contaminants due to their play behaviors, which put them in contact with many surfaces in the home [7, 21, 24–30]. In fact, Beamer et al. [31] estimated for children an average of 650 contacts per hour, with younger children exploring closer to the ground, and age driving hand-to-mouth activity [22, 24–27].

Although considerable data exists on the concentration of contaminants found in household dust (expressed in units of mass of contaminant per mass of dust), few studies explore the mass of household dust on children’s hands, making non-dietary exposure difficult to estimate accurately. Studies are also limited in capturing the various demographic characteristics of children (e.g. age difference, ethnicity, racial and social demographics, housing layouts and dynamics) which can influence play behaviors [22, 24–27]. Johnson et al. [32] has shown correlations between certain demographics (e.g., house income, family size) and dust loading which can potentially dictate how much dust a child interacts with. As a result, exploring household dust loading on hands by various socio-demographic and regional differences can help understand the potential exposures for various populations. Lastly, studies are further limited by those that also include particle sizes.

It is well established that smaller particles have a higher particle surface area (more surface-active sites per unit volume of particles), which increases the probability for contaminants to be absorbed [2, 4, 6, 10, 33, 34]. Recognizing the influence of particle size, the U.S. Environmental Protection Agency (US EPA) has established a minimum size cutoff (250 μm) for sieving soil and dust samples used in risk assessments, with a proposal to reduce this cutoff to 150 μm based on particles adhering to hands [29]. Bright et al. [3], Richardson et al. [35] and Wang et al. [34] have questioned this threshold, stating that it over-estimates the size, and, therefore, under-estimates the contaminant surface-active sites per unit volume. In fact, Siciliano et al. [36] has recommended the usage of an even smaller sieve size (45 μm) to define dust. For this reason, it is crucial to develop a methodology that can measure the particle size of dust on children’s hands for finer particle size fractions [37].

At the time of this study, 24 studies that we are aware of, document mass and/or size of particles from hands. Of these 24 studies, 8 included adult hands only [13, 23, 36, 38–42], with 1 of them focusing on indoor environments [13]. Choate et al. [42], after collecting adult hand press samples, provided particle size distribution based on sieve analysis; however, the bin sizes were wide (e.g., particles <25 μm were grouped together to provide a mass fraction). Although these size distributions are helpful in understanding a general basis of mass fraction, they do not provide a refined particle size distribution needed to evaluate overall indoor contaminant exposure in specific particle size ranges. In contrast, Cao et al. [13] did provide a more refined particle size distribution for adult hands but did not estimate dust loading.

For studies with children, we are aware of 16 studies that evaluate either dust loading or particle size distributions on hands [6, 20, 34, 43–54]. Twelve of these studies evaluate natural activities (e.g., playground, school) [6, 34, 51–54] as opposed to collecting samples from hand presses, with 4 of them evaluating solely indoor environments [45, 47, 52, 53]. Holmes et al. [45] collected samples from both adults and children after a more natural activity (e.g., work, daycare), but for these activity-based studies, no particle size range was determined. Similarly, Yamamoto et al. [54] collected hand rinses from 4-year-old children after normal outdoor activities reporting both dust loading and particle size. Their particle size distribution was obtained through laser diffraction; however, laser diffraction is not able to count particles. To address this limitation, the authors developed a regression curve between laser transmittance and amount of soil. The lower end of their regression curve corresponded to a mass of 2 mg and required extrapolation of masses less than this amount. Li et al. [48] used the same calibration curve to estimate dust loading at various environments including residential grounds. Although there are other studies that also report both dust loading and particle size on hands after normal activities [6, 20, 43, 46], these are solely focused on outdoor environments (e.g., soil, sand, clay).

These studies emphasize that well-established methods are available for the measurement of dust loading (gravimetric methods), and particle size distribution (e.g., laser diffraction, wet and dry sieving). For the collection of dust loadings directly from hands, gravimetric methods require the usage of a pre-weighed wipe, filter, or tape [20, 42, 44, 45, 47, 49–53]. Although these are easy and accurate at estimating the mass collected, obtaining the particle size distribution from the same sample is time-consuming, requiring counting and sizing samples under a microscope. Once the particles stick to the fibers of the wipe or the glue of the tape, to our knowledge, there is no proper methodology that could facilitate such measurements. As a result, to obtain particle size distribution, a secondary method would be needed.

Similarly, for particle size distributions, sieving has been effective at providing mass fractions [42]. However, sieving requires dry loose samples, which are difficult to obtain from dust adhered to hands. Ikegami et al. [46] accomplished the removal of dust adhered to hands that could potentially be used for sieving; however, they dealt with high loadings (hand presses), which may not reflect the smaller amounts of dust that adhered to hands within residential settings. Another method for particle size distribution is laser diffraction which provides a wider range of particle sizes with narrower bins [20, 34, 39, 48, 54]. Laser diffraction requires a fluid sample (e.g., hand rinses) or a dry loose sample (e.g., original soil or dust hands interact with). However, laser diffraction does not provide particle counts by size category and, therefore, cannot be used to estimate dust loading directly. Overall, if dust loading and particle size are to be measured, two different methods would be needed. To our knowledge, there is no current methodology that can provide both measurements of dust loading and particle size simultaneously.

To fill this gap, the goal of this study was to utilize a new method to measure the dust loading on children’s hands after natural indoor play activities, while also obtaining the size distribution from the same sample. This new approach directly measured the total particle volume by counting and sizing particles washed off children’s hands using a Coulter Counter, which has the sensitivity to quantitatively measure individual particles suspended in a fluid. The total particle volume was then multiplied by the density, measured from vacuumed dust samples collected from the home, and analyzed with a pycnometer, to obtain the mass. All estimated masses were then normalized by total hand surface area, and per palmar area, to obtain dust loading. This study is unique in that it is the first study, to our knowledge, that uses a Coulter Counter to estimate dust loadings and particle size from children’s hands, not requiring duplicate samples. It provides refined particle size distributions similar to laser diffraction and can potentially be used to estimate dust loadings from wipes as well. This study is also unique in that it conducts such measurements for 101 children of young ages (6 months to 6 years), inclusive of different regions of the U.S. (Greensboro, North Carolina; Miami, Florida; Tucson, Arizona), and broad social demographic backgrounds in residential settings. As a result, the data presented in this paper complements the existing literature that provides data necessary to compute children’s dermal and ingestion exposures to contaminants found in household dust.

## Materials and methods

The current study is part of a larger study (DIRT; Dust Ingestion childRen sTudy), which aimed to augment data for exposure modeling simulation [55]. Through DIRT, 450 surveys were completed to gather participating family information on demographics, children’s activities, and cleaning habits, that might contribute to household dust. All participating families had at least one child between the ages of 6 months up to 6 years and lived in or near one of three target regions (Greensboro, North Carolina; Miami, Florida; and Tucson, Arizona). A variety of strategies were used to guarantee diversity in demographics including recruiting at community events (e.g., museums, libraries, parks), posting online, as well as partnering with local organizations (e.g., elementary schools and daycares). Among the family survey participants, 101 children took part in home visits, designed to collect dust samples and document children’s activities through videography.

A home visit consisted of approximately five hours. The first hour was for setting up the cameras, as well as completing an additional in-field house survey. This additional survey involved gathering information on the surface materials of the home, size of the home (e.g., measurement of the rooms), and other variables (e.g., number of rugs, number of windows). Additionally, the height and weight of the child were measured, followed by the collection of a pre-hand rinse. At the end of the first hour, cameras would be turned on and researchers would leave the home for the family to continue their normal household routine without interruptions. After three to four hours of footage, researchers would come back into the home to complete sampling. Cameras would be turned off, followed by the sampling of a post-hand rinse, surface wipes, vacuumed dust, and soil samples. All dust samples and post-hand rinse were collected after videotaping to compare dust loadings and estimates obtained from modeling using videography data. The complete dust sample collection included vacuum socks (floors, *n* = 1 per home visit), two hand rinses (pre- and post- per child), hand traces (left and right hand per child), surface wipes (*n* = 8 per home visit), and soil samples (*n* = 2 per home visit). To promote consistency among measurements, sample collection kits were prepared by one laboratory (University of Miami). This same laboratory received all physical samples for post-processing, allowing for all analyses to be conducted using the same equipment and the same personnel. This paper focuses on the results from the vacuum socks, hand rinses, and hand traces only. More information on the larger study can be found in Ferguson et al. [55].

Age groups used in this study were based on those listed in the US EPA’s Child-Specific Exposure Factors Handbook [56] and previously defined by Firestone et al. [57]. For the breakdown of ages, 17 out of 101 children were between 6 and 12 months, 25 were 1–2 years of age, 25 were of 2–3 years of age, and the remaining 33 were between 3 and 6 years old (Table S1). One child was above the 6-year-old threshold, and, although a hand rinse was collected, it is not included in the data analysis. Additionally, out of the 101 home visits, 84 were unique houses. The difference in 17 homes was due to siblings who also participated in the visit portion of the study. Sibling participation was part of a separate home visit, where a separate set of hand rinses, vacuum sock, surface wipes, and soil samples were collected. All families that completed the survey and home visit consented to participate in the study with approved protocols (North Carolina Agricultural and Technical State University IRB #20210643) and received a total of $125 for their participation.

### Methods for field data collection

#### Hand traces collection (to be used for surface area estimates)

**Fig. 1:**
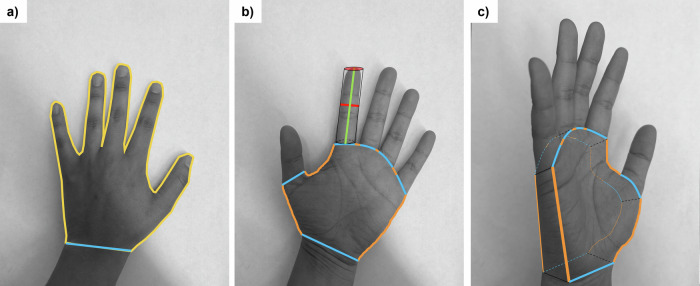
Boundary lines needed for surface area estimates. **a** Tracing boundaries for the Perone et al. [58] method (2D Surface area). Yellow line corresponds to the palmar area (palm + fingers), with a cutoff at the interstylon represented by a blue line. **b** Tracing boundaries, and line measurements needed for the Leckie et al. [59] method. Note that Leckie et al. separates the fingers from the palm to estimate surface area of the total hand. Green line corresponds to the length of the finger, red is the width of the finger (also the radius of the cylinder to be used for surface area estimates), the combination of blue and orange lines represents the palm perimeter (non-fingers), with the orange lines representing the exposure perimeter. **c** Hand at an angle to show the depth of the palm (to be estimated by the width of the middle finger) and how Leckie et al. estimates the palm surface area (non-fingers; 3D Surface Area).

Prior to hand rinses, both hands of the child were traced on a three-line graph paper with major line of 2 cm, intermediate lines of 1 cm, and minor lines of 0.25 cm. Upon receipt of the traces at the University of Miami laboratory, two methods were used to quantify the surface area of the hand. One method, described by Perone et al. [58], used ImageJ software (Wayne Rasband and Contributors, National Institutes of Health, USA; ImageJ 1.53k with Java 1.8.0_172) to digitize the traces to estimate the palmar (palm + fingers) surface area. Since the current study involved rinsing the whole hand, the second method used consisted of an additional set of measurements as described by Leckie et al. [59] and implemented by Hsi et al. [39] and Tsou et al. [51], to estimate the total hand surface area (palmar, back of hand, and exposure perimeter; Fig. 1). Additional details are provided in the supplemental text (S.1) including equations, and boundary definitions for each section of the hand. Since hand rinses were conducted using both hands, when referring to surface area throughout this paper, the sum of the left and right hand for total hand surface area is considered.

#### Vacuum sample collection (to be used for density estimates)

A vacuum sock (Midwest Filtration LLC, X-cell 100 Dust Collection Filter Sock, Cincinnati, OH, USA) was used to collect a greater amount of dust for density measurements. The pre-weighed vacuum sock was placed inside the vacuum tube, followed by inserting the mouthpiece of the vacuum inside the sock, and slightly pushing it until locked. As per standard methods [60], dust was collected in a descending back-and-forth motion for 5 min to obtain a uniform and representative sample of the entire sampled area. When available, samples were collected from a carpet or a rug (69 out of 101 vacuumed socks); if not, samples would be collected directly from the non-covered floor. After sample collection, the vacuum sock was placed inside a zip-top bag to be shipped back to the University of Miami where it was reweighed to obtain the mass of dust collected. Pre- and post-weighing utilized the same scale (Mettler Toledo ME204TE, sensitivity to 0.1 mg, Columbus, OH, USA). Samples were kept inside their corresponding zip-top bags and kept at room temperature until analysis.

#### Hand rinses collection (to be used for volume estimates, and particle size distribution)

Pre- and post-hand rinses were collected. The pre-hand rinsing began by having a parent assist in washing their child’s hands in a sink with soap (California Baby Super Sensitive Shampoo and Body Wash, USDA Certified, Los Angeles, CA, USA). Immediately after washing, the child’s hands were dried with a dust-free Kimtech wipe (Kimberly-Clark Kimtech 33330 Pure Disposable Wiper with W4 Dry, Roswell, GA, USA), followed by the transfer of their clean hands into a zip-top bag prefilled with 150 ml of background salt solution called isotone (needed for the operation of the Coulter Counter, consisting of 9 g of NaCl per Liter of Milli-Q water, filtered through a 0.2 μm filter). The child’s hands were rinsed for 20 s by having an adult hold the outside of the bag and gently rubbing the hands with the background salt solution. After the rinse was completed, the hands were dried once again with a dust free Kimtech wipe. This occurred right before any video footage was taken to provide a baseline of “clean” hands to compare to the post-hand rinse after natural play.

The post-hand rinse was used to quantify the dust loading on hands per house visit. For the post-hand rinse, hands were not washed prior to rinsing. The times for pre- and post-hand rinse collections were recorded to document the time elapsed between samples (Table S7). The average play time was of 4 hours and 14 minutes, with 75% of the recorded times being between three to four and a half hours. This can help incorporate play time as a factor in dust loading when coupled with the videography data to explore potential onloading (i.e., contact with surface) or offloading (i.e., wash or wipe events). Pre- and post-hand rinses were transferred into plastic bottles to be shipped back to the University of Miami where they were kept refrigerated until analysis.

### Methods for sample analysis—mass estimates (*m*)

**Fig. 2:**
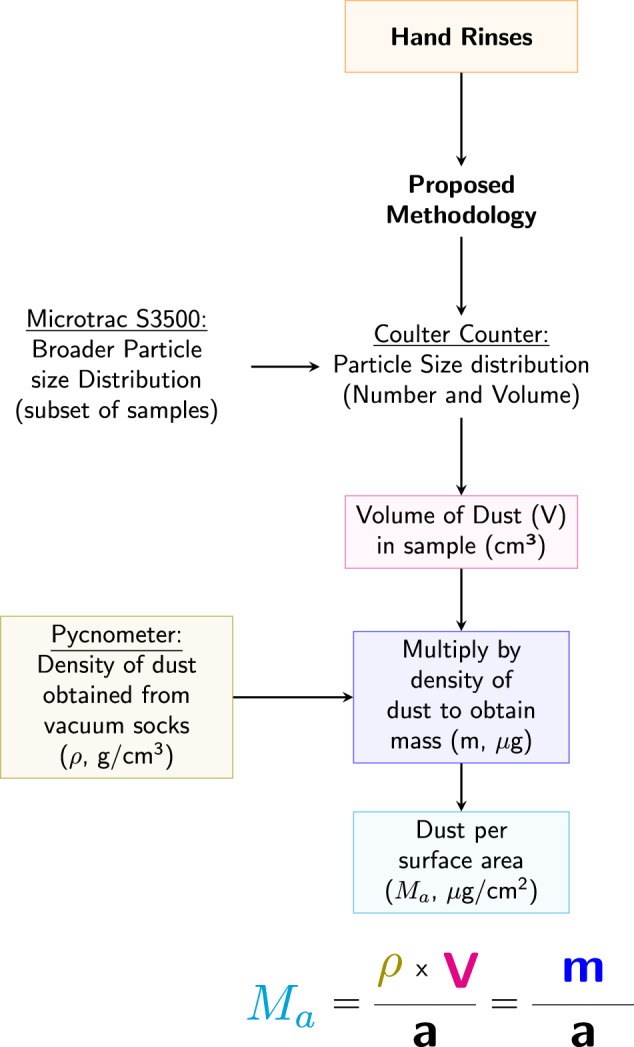
Sample analysis processes. Flow chart illustrating the process used to obtain the mass of dust per surface area for hand rinses. All measured mass values were normalized by the corresponding surface area (total hand and palmar) to obtain the dust loading.

The method to determine dust loadings is based on the product between the density of dust, *ρ*, and the volume of dust, *V*, such that *m* = *ρ* × *V*. The mass is then divided by the total hands surface area, *a*, to obtain dust loading, *M*_*a*_ (Fig. 2).

#### Pycnometer for density estimates on household dust (*ρ*, vacuum samples)

The density of vacuumed dust was measured using a pycnometer (1 ml in size) as per standard methods for each home [61]. All pycnometers were first calibrated, where the coefficient of variation (COV) for the 1 ml empty pycnometer was reported as 0.01 on average (corresponding to a standard deviation of 0.01 ml). Triplicate runs were made for those samples with enough dust available. A run consisted of weighing an empty pycnometer, followed by adding the dust sample, and then de-oxygenated milli-Q water to the bottom of the meniscus (read by human eye). The difference in weights provided the mass of the dust, and milli-Q. To estimate the volume of the milli-Q added, we multiplied the measured mass by the density of water at its current temperature. Once we had both the volume of milli-Q, and the pycnometer (obtained from calibration), the volume of dust was estimated which further allowed the density calculation. For consistency, a minimum of 0.02 g of dust was needed for a pycnometer run to occur. For quality assurance, controls were run with each batch using the ISO Standard 12103-1 Arizona Test Dust which has a known bulk density between 2.5 to 2.7 g/cm^3^. Results from control measurements were consistently within range. See supplemental text for additional details (Table S3).

To estimate the dust loading on a child’s hands, the average vacuumed dust density attained per home visit was paired with the particle volume from the hand rinse measurements of the child from that same home. For homes where the collected mass of vacuumed dust was insufficient for acceptable runs (11 out of 101 home visits), the density values were substituted with the average of the measured density for the corresponding region.

#### Coulter counter for particle size distribution and volume estimates (*V*, particles from hand rinses)

The counting and sizing of particles used by the Coulter Counter (CC; Coulter, Coulter Counter Multisizer 3, Beckman Coulter, Brea, CA, USA) is based upon the *Coulter Principle*, which measures the changes in electric resistance as a particle passes through an aperture while being suspended in an electrolyte solution (*Multisizer 3 Operator’s Manual;* Beckman Coulter Multisizer 3 Particle Characterization, Version 3.51, Beckman Coulter, Inc) [62]. The resistance created by the particle is proportional to its size. This allows for the CC to provide both the distribution of particle diameters (percentage of particles within a certain size interval or bin), and the number of particles within a given bin size interval. One limitation of the CC, however, is that counting is restricted to a window of particle diameters defined by the aperture size.

The aperture size for this study (100 μm) corresponded to a measurement window of 2 μm to 60 μm in diameter. Within this window, the CC provided two primary sets of parameters: total particle volume and diameter cutoffs. For a given amount of electrolyte (isotone) analyzed by the CC, the total particle volume of dust (area under the particle size distribution curve) can be extrapolated to the original 150 ml of the hand rinses, to obtain the total particle volume on a child’s hands, *V*. This value was then used for loading calculations. As for the diameter cutoffs, they correspond to particles whose sizes were 10% finer (D_10_), 50% finer (D_50_), and 90% finer (D_90_). For each of these cutoffs, two values were reported, one where the cutoff was defined by the number of particles (given by the additional subscript *n*), and one where the cutoff was defined by the particle volume (given by the additional subscript *v*).

Sample analysis required the CC to have low background particle levels. For this reason, a blank (20 ml of only isotone) was run prior to each sample to confirm the instrument concentrations to be below the recommended threshold range (0.5%). To optimize the counting process and minimize the chances of multiple particles going through the aperture simultaneously, samples were diluted such that their concentration was in the range of 8–14% [62]. Moreover, four drops of dispersant (Dispersant IA, Nonionic, with main component consisting of Triton X-100, Beckman Coulter, Indianapolis, IN, USA) were added. Sample volumes (20 ml mixture of hand rinse and isotone solution) were consistent across all samples and each run would last until a set number of particles had been counted (minimum of 20,000 particles). Each sample was analyzed five times, and the average distribution was used for computing the total particle number and volume, as well as the diameter cutoffs. Additional details are provided in the supplemental text (S.2; Figure S2) about sample handling and the computations made to extrapolate particle volume measurements.

#### Laser diffraction for a broader particle size distribution (Microtrac S3500)

As previously mentioned, one restriction of the CC is that its effective size range of analysis is limited by aperture size. To address this limitation, an additional set of measurements was made using a more traditional method, laser diffraction (Microtrac S3500), which is capable of analyzing a broader range of diameter sizes (0.02–2800 μm). However, since the main limitation of this technology is its inability to count particles within a set volume of water, the Microtrac was used to supplement the CC analyses by documenting the fraction of dust particles greater than the aperture cutoff size (60 μm).

The operation of the Microtrac involved flushing the system with distilled water, setting zero (particle background), and loading the sample (Microtrac FLEX Particle Size Analysis, Version 11.1.0.2, Microtrac Retsch GmbH). A sampled hand rinse was poured into the loading area (about 25 ml), until the required amount of sample (loading factor of 0.1–1 g) was reached. The sample was run three times, and the final particle distribution corresponded to the average of the three. Since the Microtrac required the use of Milli-Q water, the isotone solution required for the CC could not be used. As a result, to be able to compare results between machines, families with twins, or siblings close in age, were asked to have both of their children sampled on the same day for hand rinses (3 out of 101 houses). The child whose video camera footage was to be evaluated for that visit and used for model validation, had their hands rinsed with the isotone solution, while the sibling had their hands rinsed with Milli-Q water to be analyzed on the Microtrac. A total of three post-hand rinses were analyzed through both machines (Microtrac and CC) to obtain the diameter cutoffs for number and volume distribution (D_10_, D_50_, and D_90_). The distributions obtained were then normalized into 61 equally sized bins, ranging from 0.01 to 60 μm in diameter, to allow for the comparison of the results from both instruments.

### Statistical analysis

Descriptive statistics (average, median, standard deviation, coefficient of variation, and ranges) were computed using Excel. R studio (version 4.2.2) was used for all additional statistical analysis. Data were first evaluated for normality using the Shapiro-Wilks test. Results indicated that, except for the density values, data were not normally nor lognormally distributed. Thus, hand surface area estimates, and dust loading analysis were conducted using non-parametric tests. For variables with two categories (e.g., ethnicity, gender, left and right hand) the Mann-Whitney U test was used to compare the medians between categories. For variables that had more than two categories (age, race, region) the Kruskal-Wallis test was used instead. Since density was normally distributed, differences in mean density per region were determined using parametric tests (independent sample t-test with unpaired data). Outliers were assessed using a combination of the Rosner Test (with all data aggregated), and the Q-test (per home visit). When analyzing the Microtrac and CC particle size distributions, diameter cutoff sizes were compared using the same parametric tests.

## Results

### Total and Palmar hand surface area

The range for the total hand surface area when adding left and right hand per child was 159.5 cm^2^ to 606.4 cm^2^. The range of palmar surface area for all age groups was 59.9 cm^2^ to 210.8 cm^2^ (Table S2). In both cases (hand tracings and total hand calculations), for each child, there was no statistical difference between the left- and right-hand estimates (*p* = 0.99 for palmar, and *p* = 0.95 for total hand). This signifies that the methods proposed by Perone et al. [58], and Leckie et al. [59] were overall consistent. Additionally, the average ratio between the total hand surface area and the palmar surface area was 2.83 and was consistent across all ages. Refer to supplemental information for detailed results summarizing both the total hand and palmar surface area data by age group (Table S2).

### Density of household dust (*ρ*)

**Fig. 3:**
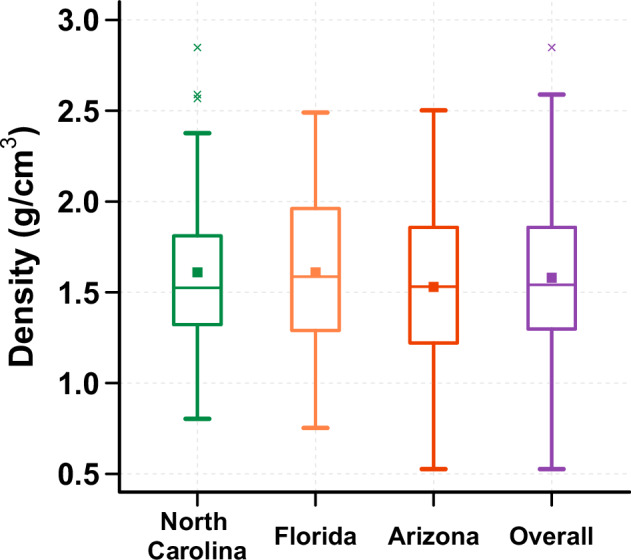
Dust density values grouped by region. The median values, identified by a central line in box, for each region were 1.52 g/cm³ for North Carolina, 1.59 g/cm³ for Florida, 1.53 g/cm³ for Arizona, and 1.54 g/cm³ for all regions combined. Additionally, the average values, identified by a square, for each region were 1.61 g/cm³ for both North Carolina and Florida, and 1.53 g/cm³ for Arizona. The dust density for all regions combined was 1.58 g/cm³.

The measured density of vacuumed household dust across all samples ranged from 0.5 to 2.8 g/cm^3^, with an arithmetic average of 1.58 g/cm^3^ (SD = 0.43) (Fig. 3 and Table S4). The average for each region was 1.61 g/cm^3^ for both North Carolina, and Florida, and 1.53 g/cm^3^ for Arizona, with no statistical differences (*p* > 0.05).

### Volume of particles on children’s hands as measured by the CC (*V)*

After extrapolation to the original 150 ml of isotone solution used for hand rinse collection, the total particle volume found on children’s hands (area under particle size distribution) ranged from 0.046 mm^3^ to 26.6 mm^3^. Overall, the particle volume in the post-hand rinses tended to be 10 times higher than the particle volume of the initial washed hands. The differences (post- versus pre-hand rinse) were of statistical significance (*p* < 0.001).

### Dust loading on hands: integration of density, volume, and surface area measurements (*M*_*a*_*)*

**Fig. 4:**
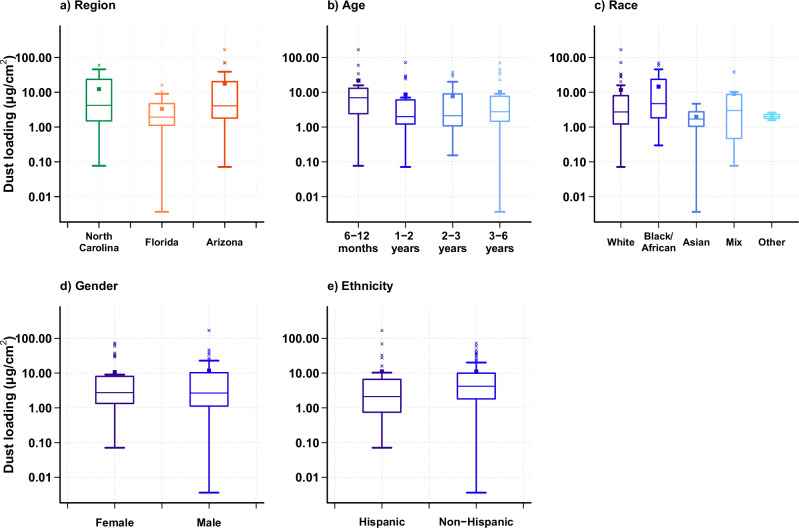
Net dust loadings (*M*_*a*_, Post Minus Pre Hand Rinse, per total hand surface area) grouped by different demographics. Boxes represent the upper and lower quartile, the error bars represent the minimum and maximum values when excluding outliers, and the × symbols outside the error bars represent outliers. The central line in the box represents the median, and the square represents the mean. **a** shows the net dust loading obtained from hand rinses (post minus pre) grouped by region, with median values shown for North Carolina (4.20 μg/cm²), Florida (1.93 μg/cm²), and Arizona (4.08 μg/cm²). **b** is grouped by EPA age categories with median values for 6 ≤ age < 12 months (6.98 μg/cm²), 1 ≤ age <2 years old (1.99 μg/cm²), 2 ≤ age <3 years old (2.12 μg/cm²), and 3 ≤ age <6 years old (2.76 μg/cm²). **c** is grouped by race with median values for White (2.71 μg/cm²), Black/African American (4.72 μg/cm²), Asian (1.70 μg/cm²), mix race (3.01 μg/cm²), and others (1.99 μg/cm²). **d** is grouped by gender with median values for female (2.73 μg/cm²) and male (2.67 μg/cm²). **e** is grouped by ethnicity with median values for Hispanics (2.12 μg/cm²), and Non-Hispanics (4.19 μg/cm²).

From the product of the particle volume and dust density, the accumulation of dust (post- minus pre-hand rinse) during the three to four hours of playing time ranged from 0.001 mg to 39.7 mg. When normalized per total hand surface area, the dust loadings ranged from 0.004 μg/cm^2^ to 167.6 μg/cm^2^, with an overall median of 2.72 μg/cm^2^. When evaluated by region, North Carolina had the highest median dust loading on children’s hands (4.20 μg/cm^2^) followed by Arizona (4.08 μg/cm^2^) and Florida (1.93 μg/cm^2^), with no statistical difference among regions (*p* > 0.05; Fig. 4). Additionally, for the current study, the 6–12 months age group had the highest median dust loading (6.98 μg/cm^2^), while the 1-to-2-year-old children had the least (1.99 μg/cm^2^); however, these differences were not statistically significant. Similarly, no statistical difference was observed by ethnicity, race, nor gender (*p* > 0.05) (Fig. 4).

Given that the ratio of the total hand to palmar surface area is 2.83, the ratio of the corresponding dust loading when normalized by the total hand area versus the palmar area is 0.35 (inverse of 2.83). Meaning that, for every gram of dust per palmar area, there are 0.35 g of dust per total hand area. This would bring the range of dust loadings normalized per palmar area from 0.01 μg/cm^2^ to 475 μg/cm^2^. The dust loadings presented can be further used to compute children’s dust dermal contact and ingestion rates for contaminant exposure analysis. See supplemental information for detailed results summarizing dust loading with the post-minus pre-hand rinse for the total surface area and the palmar surface area, respectively (Tables S5 and S6). Additionally, the complete data set for all children (without subtraction of the pre-hand rinse) is also available in the supplementary text (Table S7).

### Particle size distribution from dust on children’s hands

**Fig. 5:**
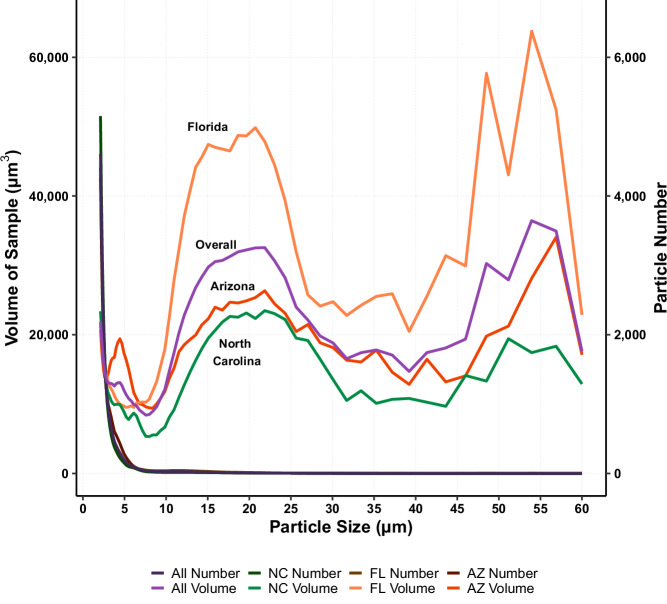
Overlay of net particle size distributions from post-hand rinses collected across three regions (North Carolina [NC], Florida [FL], and Arizona [AZ]) as measured by the CC. Darker color lines (right axis) are distributions by particle number (all lines overlap) and colored lines (left axis) are distributions by particle volume. Difference in shape is attributed to the number of particles counted within a bin. Smallest bin size is between 2.00 μm and 2.109 μm, with the largest bin size being between 56.89 μm and 60.00 μm.

As analyzed by the CC, particles of dust obtained from children’s hands can be described by volume and number distribution. When evaluating the particle distribution by number, the primary peak occurs at the lowest threshold of particles with 2 μm in diameter (Fig. 5—darker lines). The median particle diameter (D_50,n_) was measured at 2.49 μm, with the majority of particles (D_90,n_) being less than 3.91 μm in diameter (Table 1). No statistical difference were observed when grouping data by region nor demographics. When comparing the subset of hand rinses that were analyzed by both the Microtrac (laser diffraction) and the CC, the number distribution was consistent between both machines with 99.9% of the particles, by count, being <60 μm (CC aperture upper limit). In fact, the diameter cutoff sizes (D_10,n_: Microtrac = 1.74 μm, and CC = 2.07 μm; D_50,n_: Microtrac = 2.53 μm, and CC = 2.93 μm; D_90,n_: Microtrac = 5.74 μm, and CC = 5.04 μm), and mean diameters (Microtrac = 3.35 μm, and CC = 2.93 μm) showed no significant difference (*p*  >> 0.05).

**Table 1:** Average particle diameter obtained from post-hand rinse grouped by state analyzed through the CC.

City	*N*^a^	Mean	Std. Dev.	D_10_	D_50_	D_90_
Number Distribution Diameter Sizes (*D*_n_, μm)						
North Carolina	34	2.57	1.32	2.06	2.38	3.53
Florida	32	2.76	1.4	2.11	2.48	4.19
Arizona	33	2.82	1.36	2.14	2.63	4.04
Overall	99	2.72	1.36	2.10	2.49	3.91
Volume Distribution Diameter Sizes (*D*_v_, μm)						
North Carolina	34	11.10	2.58	2.84	13.73	34.81
Florida	32	12.92	2.39	3.87	15.46	35.18
Arizona	33	11.26	2.44	3.21	13.26	34.36
Overall	99	11.74	2.47	3.30	14.13	34.78

D_10_ is defined as the size diameter where 10% of the particles in the sample were found below that threshold. The same definition applies to D_50_ (50%) and D_90_ (90%).

^a^North Carolina and Florida had one house with no collection of post-hand rinse.

As a contrast to the number distribution, the volume distribution, as analyzed by the CC, consisted of a primary peak for particles between 10 and 30 μm in diameter, with a secondary peak beginning around 45 μm (Fig. 5—colored lines). Fifty percent of the particles (D_50,v_) had a diameter <14.13 μm (median), with the majority of particles (D_90,v_) being <34.9 μm (Table 1). When comparing the volume distribution from the subset of hand rinses analyzed by the Microtrac and the CC, particles <60 μm (CC aperture upper limit) represented, on average, only 49% of the broader distribution (Microtrac has an upper range of 2800 μm in diameter). In fact, the mean volume diameter between both machines (Microtrac = 74.81 μm, and CC = 17.31 μm) was statistically different (*p* < 0.001). As for the cutoff sizes, D_10,v_ (Microtrac = 11.09 μm, and CC = 6.28 μm) showed no significant difference (*p* > 0.05), while D_50,v_ (Microtrac = 60.96 μm, CC = 20.01 μm) and D_90,v_ (Microtrac = 159.93 μm, CC = 35.59 μm) were significantly different (*p* < 0.001).

## Discussion

The purpose of this study was to estimate dust loadings from children’s hands, while also obtaining the particle size of the dust after natural indoor play activities. The hand dust loadings computed (0.004–167.6 μg/cm^2^ for total hand surface area) were consistent with some previous studies, including those focused on palmar area [44, 53] and total hand area [45, 48, 51, 54]. For example, Holmes et al. [45] estimated a range of 7.3–150 μg/cm^2^ soil loading for children <6.5 years old when using the total hand surface area. In contrast, other studies report higher dust loading values; this difference could be due to the methods used (palm presses) as well as the soil type (e.g., outdoor soil, sand, and clay) [20, 42, 43, 47, 49]. Hsi et al. [39] have mentioned the dependence between soil loading and soil type, with other studies establishing the importance of moisture [23, 38, 40]. This study does not evaluate moisture, and the loadings were representative of indoor household environments only. Additionally, the wider range of values in the current study, especially on the low end, may be due to the usage of the Coulter Counter which provided a lower detection limit on our mass estimates (0.001 μg).

With respect to density, Hunt et al. [63] categorized household dust as having two fractions: one consisting of particles whose density was greater than 1.9 g/cm^3^ (sinkers), and a secondary fraction of particles with a density less than 1.9 g/cm^3^ (floaters). Sinkers tended to be mineral grains, paint particles, and outdoor soil, while the floaters consisted of fibers, residues, food debris, and organics. Other studies have even estimated the density of hair (ranging from 0.59 to 2.39 g/cm^3^) which was commonly found in vacuumed dust samples [64, 65]. Considering that the samples in this study were not sieved prior to analysis, the wide range of density values obtained is likely associated with the difference in composition of household dust.

In regard to the particle size, previous studies have stated that the median (D_50,v_) of particles adhering to hands tended to be <65 μm in diameter [3, 13, 35, 36, 46, 48, 50, 54]. For example, Yamamoto et al. [54] reported a D_50,v_ of particles adhering to children’s hands of 39 ± 26 μm in diameter, while Li et al. [48] found a D_50,v_ of 28.5 μm among preschoolers. The results from the current study were about half of those reported. This difference is likely attributed to the CC aperture upper size (60 μm) skewing the particle distribution towards smaller particles by a factor of about 50%, as well as the type of soil (outdoors versus indoors). Although Li et al. [48] did include samples from residential areas, Yamamoto et al. [54] solely focused on outdoor soil. Similarly, Cao et al. [5] evaluated indoor environment (office dust) estimating that 50% of particles adhering to adult hands had a particle diameter <25 μm. Other studies have documented much larger particle diameters (>400 μm); however, these studies are only reflective of outdoor environments, like sand [20, 43]. The type of soil can greatly influence the size of a particle (e.g., fine dust vs. sand), and as a result also the mass.

Previous studies have established that particles <60 μm represent only a fraction (25–46%) of the mass of dust adhering to both surfaces and children’s hands [13, 14, 42, 46, 48]. The current study was consistent with particles <60 μm representing less than 50% of the total mass. When considering the particles by count some studies have defined the majority of particles (D_90,n_*)* to be <100 μm [2, 39, 46, 48]; however, Duggan et al. [6] have established a lower D_90,n_ of 10 μm, with an average of particles adhering to hands of 4.5 μm in diameter. The 4.5 μm is consistent with the results of the current study which found D_90,n_ of 3.91 μm. This signifies that, although the smaller range of particles might not contribute the most to the total volume, they do dominate in numbers. Additionally, it has been previously established that household dust consists of particles that are in constant resuspension, due to indoor activities, forming a unique composition of multilayers [66–68]. The small fraction of particles on children’s hands could be attributed to settled dust from upper layers consisting of finer particles.

Additionally, several studies have documented the significance of particle size in evaluating exposure to contaminants, where the concentration of contaminants tends to increase as particle size decreases, especially in metals [2, 6, 8, 10, 17, 36]. This has led other researchers to recommend a smaller size range of particles (<45 μm) to be used for risk assessments due to their higher probability of adhering to hands and their larger surface area per unit volume, which can potentially accumulate more contaminant [3, 4, 13, 14, 36]. In fact, Cao et al. [69] addressed this concern by recalculating data from a previous study. The method applied, formulated by the authors, separated household dust into two main categories (non-adherent fractions and adherent fractions), which were further divided into subcategories representing different particle sizes for exposure risk estimates (25–500 µm, <2 mm, <500 µm, <250 µm, <150 µm, <53 µm, <25 µm, <4 µm). Their results showed that when comparing the exposure estimates from the highest fraction (<500 µm) to the smallest fraction (<4 µm) the concentrations had a 10-fold difference. For example, carbaryl, an insecticide which causes neurological effects [70], had a concentration of 0.47 µg/g for particles 25-500 µm in diameter, which increased to 4.6 µg/g when analyzing particles <4 µm in diameter. When comparing the surface area of the D_90,v_ (35 μm) measured in this study to the current U.S. EPA size cutoff (250 μm) for sieving soil and dust samples in risk assessment [29, 56], there is a 615% increase in particle surface area per unit volume. Assuming that contaminant adsorption is proportional to the surface area of the particle, using the current cutoff suggests a large underestimation of exposure when assessing contaminant concentrations in dust. The results obtained through this study (D_50,n_ = 2.49 μm and D_50,v_ = 14.1 μm) further supports that a smaller cutoff size should be used. Future work and risk assessments should document contaminant concentrations in smaller size fractions and their impacts on exposure.

### Limitations

In regard to the collection of hand rinses, one of the limitations was children’s cooperation, or lack thereof, needed to obtain a representative hand rinse. Several children in the study, especially children less than 2 years old, did not feel comfortable rinsing their hands within the zip-top bag. There were children who only allowed their parents to rinse their hands. Both scenarios might have contributed to the variability of results since rinsing may not have been as thorough. A secondary limitation is the possibility of dust agglomeration since no dispersant was added right after sampling. Yamamoto et al. [54] showed that hand rinses without dispersant caused the particle size distribution to shift towards the larger particles. Considering that the CC requires its own dispersant as part of its standard procedure, future studies are recommended to add the dispersant on the day of collection instead of on the day of analysis to minimize particle agglomeration. A related limitation on particle measurements is the possibility that the size of the particles reported may not reflect those found on a child’s dry hands due to the adsorption of the isotone solution during the hand rinsing step. However, by using the isotone, the CC moisture gained should not impact the resistivity measure used to size the particles providing some degree of standardization. More work is recommended to evaluate the impacts of moisture on the size of the particles on dry hands versus those released into the CC isotone solution during washing.

A third limitation is the CC aperture. As mentioned, the CC aperture window allowed for the analysis of particles between 2 and 60 μm in diameter. When compared against the Microtrac results, a few larger particles, beyond the 60 μm threshold, were shown to significantly impact the diameter cutoffs obtained in this study, given the tendency of larger particles to skew the volumetric distribution. As a result, this could lead to an underestimation of the dust loadings and particle count presented in this study. One method to approach this is by using a larger aperture that can provide a wider window of measurements. For example, the CC also has a 400-μm aperture which corresponds to a particle size window of 8 μm to 240 μm in diameter. This study was not able to apply this method; however, it would be interesting to compare the dust loadings obtained in this study to those when using a larger aperture on the CC. Lastly, the CC assumes all particles to be of spherical shape, which is not accurate for household dust that includes elongated particles like fibers, and skin flakes. These types of particles would not have a volume equal to that of a sphere and could lead to an overestimation in the total particle volume, which can further affect the dust loadings calculated.

To directly compare the mass estimates between traditional gravimetric methods and the CC method, a few wipes used for dust sampling (*n* = 3), were analyzed using the same protocol as for hand rinses. These wipe samples with known mass were placed inside a zip-top bag containing 150 ml of isotone where they were gently rubbed for 5 min to release the particles into the fluid. The particle number and sizes were then measured using the CC and the results were then multiplied by the estimated dust density. On average, we recovered 73% of the known mass value. The remaining 27% could have been due to incomplete removal of the dust particles from the fibers of the wipe, the coulter counter aperture not allowing larger particles to be counted, or the influence of the previously discussed limitations. We recommend further analysis to verify this accuracy.

### Recommendations

The approach developed through this study provides a new alternative for the analysis of dust from children’s hands. Our study found that dust loading was impacted significantly by the presence of large particles, whereas smaller particles dominate the contribution towards the surface area of the particles where contaminants can accumulate. Currently, guidelines provided by the US EPA Exposure Factor Handbook [56] do not yet account for the impact of particle size on exposure estimates. The only recommended threshold is the one used for lead sampling which considers sieving for particles <250 µm in diameter [29]. Based on the results obtained in this study, we believe this threshold must be reevaluated to a smaller one (<40 µm). Additionally, Cao et al. [69] emphasized the importance of particle size by providing a new methodology for estimating human exposure. The methodology is composed of three steps: 1) analyze adherent dust fractions for chemical concentrations, 2) compute mass of chemical by particle size by multiplying the concentration by its corresponding mass fraction, and 3) determine daily exposure as the sum of the chemical mass from each size fraction. The results from the current study correspond to the first step by providing additional data for the low end of dust loadings and particle size distributions that adhere to hands.

## Conclusions

The current study used a new approach to quantify the amount of dust found on children’s hands after natural play activities. Overall, dust loadings on a total hand surface area basis were 11.13 μg/cm^2^ on average (median 2.72 μg/cm^2^) with a range from 0.004 μg/cm^2^ to 167.6 μg/cm^2^. Although there are possible factors affecting dust loadings, no statistical differences were observed between regions, gender, age, race, nor ethnicity. In regard to particle size, the volume distribution obtained from children’s hands showed that the majority of particles were <35 μm in diameter (D_90,v_), with the majority of individual particles by count (D_90,n_) to be <4 μm. However, these small particles represent only a fraction of the total mass. This was consistent across all three regions. As a result, current guidelines for soil and dust sampling that recommend sieving (<250 μm) may overestimate dust particle sizes found on children’s hands, thereby potentially underestimating children’s exposure to contaminants which are enriched in the smaller size fractions. Future work should differentiate between the mass and particle size that is found on the hands versus what is ingested to further understand contaminant exposure. In fact, the dust loadings presented here can be used for model validation, especially since the post-hand rinse collected is equivalent to how much dust adheres to a child’s hands after a certain period of playing time. We recommend combining the results obtained herein with exposure modeling to estimate ingestion rates and consequently risks to children from residential dust.

## Supplementary information

Supplementary Text

## Data Availability

All data generated or analyzed during this study are included in this published article and its supplementary information files.
