# Supplementary material for: Mass and particle size distribution of household dust on children’s hands
Source: J Expo Sci Environ Epidemiol. 2025 Feb 10;36(1):41–51. doi: 10.1038/s41370-025-00749-3 (PMC12795756; doi:10.1038/s41370-025-00749-3)
Supplement: Supplementary file 1 — Supplementary Text [file 41370_2025_749_MOESM1_ESM.docx]

**SUPPLEMENTAL TEXT FOR**

**Mass and Particle Size Distribution of Household** **Dust on Children’s Hands**

Cristina Fayad Martinez^1^, Maribeth Gidley^1^, Matthew A. Roca^1^, Ryuichi Nitta^1^, Ali Pourmand^2^, Arash Sharifi^2,3^, Foluke Adelabu^4^, Jenna Honan^5^, Olusola Olabisi Ogunseye^5^, Paloma Beamer^5^, Helena Solo-Gabriele^1^, Alesia Ferguson^4^

^1^ Department of Chemical, Environmental, and Materials Engineering, University of Miami, Coral Gables, FL 33146. USA

^2^Neptune Isotone Laboratory, Rosenstiel School of Marine, Atmospheric, and Earth Science, University of Miami, Miami, FL, 33149.

^3^Isobar Science, Research and Development Department, Miami, FL, 33155.

^4^Department of Built Environment, North Carolina Agricultural and Technical State University, Greensboro, NC 27411, USA

^5^Department of Community, Environmental and Policy, Mel and Enid Zuckerman College of Public Health, University of Arizona, Tucson, AZ 85724, USA

For consideration for potential publication in:

*Journal of Exposure Science and Environmental Epidemiology*

Version dated: January 8, 2025

| **Supplemental Table S1. Demographics of Children that Participated In House Visits.** | | |
| --- | --- | --- |
| Individual Variables | | *N* |
| **Region** |  | |
| North Carolina | | 35 |
| Florida | | 33 |
| Arizona | | 33 |
| **Age** | |  |
| 6 ≤ age < 12 months | | 17 |
| 1 ≤ age < 2 years | | 25 |
| 2 ≤ age < 3 years | | 25 |
| 3 ≤ age < 6 years | | 33 |
| age > 6 years | | 1 |
| **Gender** | |  |
| Female | | 49 |
| Male | | 51 |
| Unknown | | 1 |
| **Race** | |  |
| White | | 50 |
| Black or African American | | 26 |
| Asian | | 6 |
| Mix | | 15 |
| Other | | 4 |
| **Ethnicity** | |  |
| Non-Hispanic/Latino | | 61 |
| Hispanic/Latino | | 39 |
| Unknown | | 1 |
| **Overall** | | 101 |

**S.1. Calculations for the surface area of the palmar and total hand area.**

Two main methods were used for the estimation of surface area. First, the method proposed by Perone et al. (2021) was used to estimate only the palmar area which consists of the palm (non-finger surface area) and all fingers (Figure S1 a, b; yellow and black line), with a cutoff at the interstylon (wrist – blue line) (Figure S1). Perone’s method consisted of tracing the hand on a three-line paper of major lines of 2 cm, medium lines of 1 cm, and minor lines of 0.5 cm. This image was digitized and uploaded into ImageJ for palmar surface area estimation.

Since for this study both hands were completely rinsed, to normalize our dust masses we also estimated the total hand surface area as proposed by Leckie et al. (2000), and previously used by Hsing-Cheng et al. (2018). The total hand consisted of the palms (non-finger surface area), back of hands without fingers, all ten fingers, and the exposure perimeter of both hands (thickness of hand, and in between fingers) (Figure S1 and Figure 1 in main text).

In the case of Leckie’s method, there are certain assumptions to consider:

1. Fingers are to be treated as cylinders, where the diameter of the cylinder is the width of the finger. The surface area is the sum of the lateral surface area plus the area of one of the circular faces. The surface area of the circle face represents the tip of the finger.
2. The thickness of the palm is defined by the width of the middle finger of each hand.

As a result, fingers will be estimated individually and separately from the palmar area (Figure S1). To estimate the Finger Surface Area (*FSA*) for finger *i*:

$${FSA}_{i}=\left( \pi\cdot{FW}_{i}\cdot{FL}_{i} \right)+\frac{1}{4}\left( \pi{FW}_{i}^{2} \right)$$

Where *FW_i_* is the finger width (Figure S1 b; red lines), and *FL_i_* is the finger length (Figure S1 b; green lines). Once the area is estimated for each of the fingers, the Total Finger Surface Area is computed as:

$$TFSA= \sum_{i=1}^{5} {FSA}_{i}$$

As for the palmar area, in contrast to the Perone et al. (2021) method where the palm includes all five fingers, for the Leckie et al. method the palm consists of all non-finger surface area (Figure S1 c, and d). To be able to estimate this part of the hand, Leckie et al. (2000) recommends to first estimate the palm exposure perimeter (*PEP*). The *PEP* is related to the expose side of the palm which corresponds to the surface area of the side of the palm that is not attached to fingers or wrist (Figure S1 d; orange lines). To estimate *PEP* the following equation is used:

$$PEP=TPP- \left( \sum_{i=1}^{5} {FW}_{i}+WW \right)$$

Where *TPP* is the total palm perimeter (Figure S1 c, and d; combination of orange and blue lines), *WW* is the wrist width (Figure S1 b; bottom blue line), and *FW* is the finger width of each of the five fingers (Figure S1 b; red lines). Once the *PEP* is estimated, the total palm surface area for one hand is computed using the following equation:

$$TPSA= \left( 2\times PSA \right)+MFW\times PEP$$

Where, *PSA* is the palm surface area as defined by Leckie et al. (2000) without the inclusion of the fingers, *MFW* the middle finger width, and the *PEP* is the palm exposure perimeter. The surface area used to estimate dust loadings included the sum of both the left and right hand for both the Perone and Leckie method.


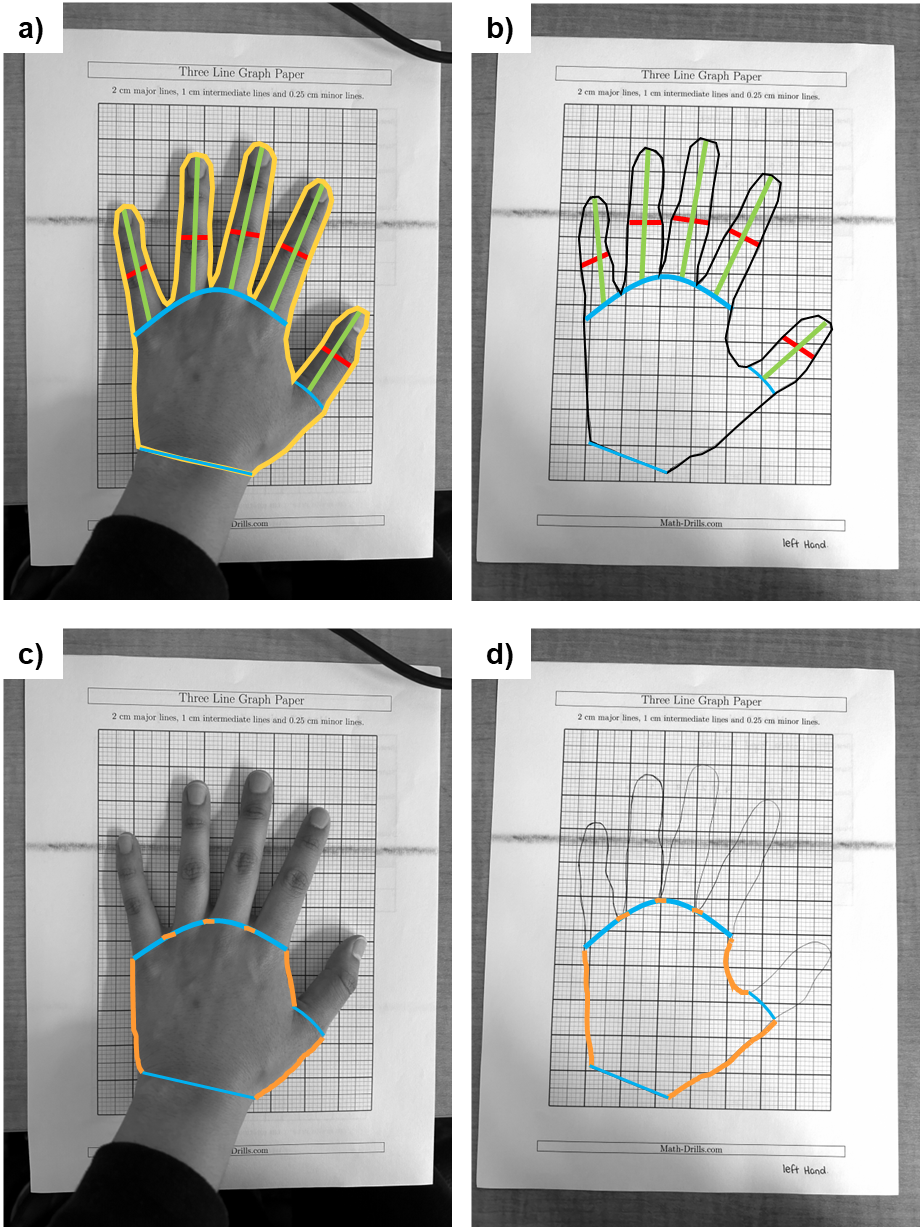


**Figure S1. Cutoff tracings for palm and total hand surface area measurements.** a) Picture of hand before tracing. Yellow line corresponds to the whole palmar area (surface of palm + fingers) used for Perone et al.’s (2021) tracing method. The blue line corresponds to the cutoff of the palm (non-fingers), where the bottom blue line is the interstylon. Green lines correspond to the length of a finger, with the red lines being the width of each finger (center of green line) used for Leckie’s method, b) trace of hand with measurement lines. Black line corresponds to the whole palmar area (palm + fingers), c) picture of hand before tracing with added orange lines to represent the palm exposure perimeter, d) trace of hand with second set of measurement lines.

| **Supplemental Table S2.** Average of Palmar and Total Hand Surface Area Grouped by Age [cm^2^] | | | | | | | | |
| --- | --- | --- | --- | --- | --- | --- | --- | --- |
|  |  | **Palmers** | | | **Total Hands** | | | |
| **Age Group** | *N* | Palmers* | Std. Dev. | Coeff Var. | Total Hands* | Std. Dev. | Coeff Var | Ratio** |
| 6 ≤ age < 12 months | 17 | 77.14 | 9.48 | 0.123 | 210.89 | 37.91 | 0.18 | 2.73 |
| 1 ≤ age < 2 years | 25 | 89.58 | 11.95 | 0.133 | 255.00 | 37.54 | 0.15 | 2.85 |
| 2 ≤ age < 3 years | 25 | 110.95 | 16.43 | 0.148 | 315.55 | 54.58 | 0.17 | 2.84 |
| 3 ≤ age < 6 years | 33 | 143.35 | 24.01 | 0.167 | 405.44 | 77.80 | 0.19 | 2.83 |
| age > 6 years | 1 | 205.91 | - | - | 606.38 | - | - | 2.95 |
| **Overall** | 101 | 111.49 | 32.29 | 0.290 | 315.20 | 97.53 | 0.31 | 2.83 |
| *The surface area presented corresponds to the sum of the areas of both left and right palms/hands. For Palmar (sum of both palms) and Total hand (sum of both hands) calculation details see section S.1.  **Ratio of the total hand area divided by the palmar area. | | | | | | | | |

**S.2 Calculations for Volume Estimates from Children’s Hands**

To determine the particle volume estimates on children’s hands, a Coulter Counter (CC) was used to count and size the particles collected through hand rinses. The CC relies on the *Coulter Principle* which measures the changes in electrical resistance as a particle passes through an aperture. This change in resistance is proportional to the particle diameter. In order to measure the electrical resistance, samples must be diluted in an isotone solution (9 g of NaCl per Liter of Milli-Q water, filtered through a 0.2 μm filter), that allows the sample to carry an electric charge. As a result, all hand rinses were collected using the isotone solution instead of Milli-Q water.

During analysis, to optimize the counting process and minimize the changes of multiple particles going through the aperture simultaneously, hand rinses needed a concentration of particles within the range of 8-14% (Multisizer 3 Operator’s Manual). For this reason, although some hand rinses had very little particles and did not require dilution, other hand rinses had to be diluted to obtain the recommended threshold before analysis. Dilution of hand rinses for CC runs consisted of the following:

1. Placing an empty ACCUVETTE® (CC 20 ml beaker) on the weighing scale and recording the weight of the empty container
2. Adding 10-20 ml of isotone solution into the ACCUVETTE®. The amount added was dependent on the concentration of the original hand rinse. For instance, if the concentration given by the CC was 20%, then a dilution of 50-50 (10 ml of isotone and 10 ml of hand rinse) was needed to lower concentration to 10%. This dilution was accomplished by first pipetting the appropriate volume of isotone into the ACCUVETTE®
3. The weight of the ACCUVETTE® and the isotone was recorded
4. With the usage of another pipet, the remaining milliliters needed for the dilution were transferred from the hand rinse into the ACCUVETTE® containing the isotone. For example, if 10 ml of isotone was added, then 10 ml of the hand rinse were added. All final volumes of diluted samples (20 ml) were consistent across analysis.
5. The weight was once again recorded (ACCUVETTE® + isotone + hand rinse)
6. Finally, an additional 4 drops of dispersant agent (Dispersant IA, Nonionic, with main component consisting of Triton X-100) were added into the 20 ml prepared sample. The weight was recorded one last time (ACCUVETTE® + isotone + hand rinse + dispersant). The combination of the isotone + hand rinse + dispersant is referred to as the “prepared sample”
7. The ACCUVETTE® was then closed to be able to gently mix the prepared sample by turning it around a few times.
8. The concentration of the prepared sample was then verified through the CC to be within the required range (submerging of aperture into solution) using the preview setting. If the concentration was still high, the process would be repeated with a different dilution until the threshold was met.
9. The prepared sample was then analyzed through the CC by submerging the aperture into the solution and clicking start. Each run lasted until a minimum of 20,000 particles had been counted. Once the run ended, the program would automatically flush the aperture.
10. Before initiating another run, the ACCUVETTE® with the remaining prepared solution was reweighed without cap on. The difference in weight prior to a run and after a run represented the amount of prepared sample that passed through the aperture
11. Each prepared sample was analyzed and reweighed a total of five times.
12. The CC provides both the particle size distribution (by number, surface area, and volume), and the amount of particles counted, number, total surface area, and total volume (area under the curve) that was analyzed.

Figure S2 displays the calculations done to extrapolate the total volume of particles within a hand rinse consisting of 150 ml of isotone solution. This process was the same for all hand rinses.


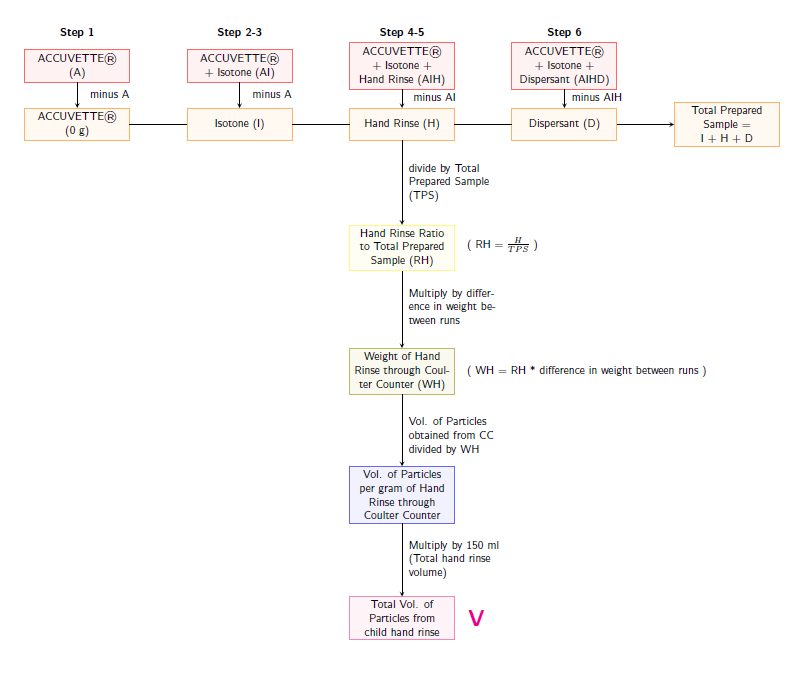


**Figure S2. Flow chart illustrating calculations for volume estimates.** Note that if hand rinses did not require dilution, then no isotone would be added, and the total prepared sample would only consist of the hand rinse and dispersant. The step numbers correspond to the above list describing the procedure for sample preparation.

**S.3 Summary of Quality Control Measurements**

The quality control tests evaluated include: 1) accuracy and precision of dust density measurements, 2) accuracy and precision of dust mass calculations, 3) particle size distribution of standards, and 4) blanks

**Dust Density:** The accuracy was evaluated by measuring a control of known density (Arizona Test Dust, Powder Technology Inc, with values ranging between 2.5 to 2.7 g/cm^3^). This control was analyzed once after every analyzing day to obtain a measure of precision using a 1 ml pycnometer.

**Dust Mass:** The Arizona Test Dust was analyzed in-house using laser diffraction (Microtrac S3500) as well as the Coulter Counter using the same protocol established for the hand rinses. In both cases, the samples analyzed consisted of 150 ml of fluid (Milli-Q water for the laser diffraction, and background isotone solution [9 g of NaCl per Liter of Milli-Q water] for the Coulter Counter) inside a zip-top bag with a fixed amount of Arizona Test Dust (0.1 to 1 g). Using the method proposed, the ratio between the dust mass estimated to the known fixed value added was on average 1:4.

**Particle Size Distribution of Standards:** For particle size via the Coulter Counter, standards included a L10 standard (particles of 10 μm in diameter). Once a week, the 10 μm standard was measured to guarantee accuracy in results.

**Blanks:** A blank consisted of only 150 ml of fluid (Milli-Q water for the Microtrac S3500, and background isotone solution [9 g of NaCl per Liter of Milli-Q water] for the Coulter Counter) with no added dust. For the Coulter Counter, a blank was analyzed five times using the same protocol as the one proposed for the hand rinses. The average mass calculated for a blank was of 175 μg, with a lowest value of 77.1 μg. Seventy-three out of 102 pre-hand rinses were below detection limits indicating good washing of the hands.

**Supplemental Table S3.** Summary of Quality Control Standards.

| Variable | *N*^a^ | Known Value | Obtained Value | Standard Deviation | Coefficient of Variation |
| --- | --- | --- | --- | --- | --- |
| Density through Pycnometer  (Arizona Test Dust) | 12 | 2.5 – 2.7 g/cm^3^ | 2.54 g/cm^3^ | 0.06 | 3% |
| Microtrac Particle Distribution  (*MV*^b^, Arizona Test Dust) | 21 | 25.36 μm in diameter | 18.05 μm in diameter | 2.10 | 12% |
| Microtrac Particle Distribution  (*D_50,v_* Arizona Test Dust, without adjustment) | 21 | 14.16 μm in diameter | 10.58 μm in diameter | 0.89 | 8% |
| Coulter Counter Particle Distribution  (*D_50,v_* Arizona Test Dust, with adjustment) | 40 | 12.04 μm in diameter^c^ | 11.3 μm  in diameter | 2.05 | 27% |
| Coulter Counter Standard Size Standard L10 | 20 | 10.14 μm in diameter | 10.5 μm  in diameter | 1.36 | 5% |

**^a^***N* corresponds to the total number of runs done in lab to estimate the obtained value.

**^b^**MV is the mean diameter (μm) of the volume distribution as measured by the Microtrac S3500.

**^c^**The adjusted value of *D_50,v_* is from the Arizona Test Dust known distribution obtained through the Microtrac S3500 provided by the company Power Technology Inc., (PTI, 2020).

| **Supplemental Table S4.** Average density values of dust (g/cm^3^) measured with a 1 mL pycnometer | | | | | | | |
| --- | --- | --- | --- | --- | --- | --- | --- |
| City | N* | Mean | Median | Min | Max | Std. Dev | Coeff. Var |
| North Carolina | 83 | 1.61 | 1.52 | 0.80 | 2.85 | 0.41 | 26% |
| Florida | 56 | 1.61 | 1.59 | 0.75 | 2.49 | 0.44 | 27% |
| Arizona | 82 | 1.53 | 1.53 | 0.53 | 2.50 | 0.43 | 28% |
| Overall | 221 | 1.58 | 1.54 | 0.53 | 2.85 | 0.43 | 27% |
| *Corresponds to the total number of runs corresponding to the city, including all houses (maximum of three pycnometer runs per home) | | | | | | | |

| **Supplemental Table S5.** Net Dust Loading from Hand Rinses (Post minus pre-hand rinse) normalized by palmar surface area. | | | | | | | | | | | |  |
| --- | --- | --- | --- | --- | --- | --- | --- | --- | --- | --- | --- | --- |
|  | *N*** | Mass per Palmar Surface Area (μg/cm^2^) | | | | | | Std. Dev. | | | Coeff. Var. | |
|  |  | Mean | Median | | Min | | Max | |  |  | | |
| **Region** | | | | | | | | | | | |  |
| North Carolina | 28 | 34.45 | 12.20 | BDL* | | 176.87 | | 46.41 | | | 1.35 | |
| Florida | 30 | 9.37 | 5.58 | BDL* | | 46.11 | | 10.39 | | | 1.11 | |
| Arizona | 30 | 50.44 | 11.17 | BDL* | | 475.01 | | 97.24 | | | 1.93 | |
| **Age** | | | | | | | | | | | |  |
| $6 \leq age<12$months | 15 | 58.91 | 19.36 | BDL* | | 475.01 | | 123.1 | | | 2.09 | |
| $1 \leq age<2$years | 23 | 24.87 | 6.00 | BDL* | | 209.25 | | 47.51 | | | 1.91 | |
| $2 \leq age<3$years | 23 | 21.92 | 6.00 | 0.44 | | 113.86 | | 32.34 | | | 1.48 | |
| $3 \leq age<6$years | 27 | 29.61 | 7.01 | BDL* | | 192.77 | | 48.87 | | | 1.65 | |
| **Gender** | | | | | | | | | | | |  |
| Female | 45 | 30.55 | 7.87 | BDL* | | 209.25 | | 52.17 | | | 1.71 | |
| Male | 42 | 32.56 | 7.27 | BDL* | | 475.01 | | 76.69 | | | 2.36 | |
| **Race** | | | | | | | | | | | |  |
| White | 43 | 33.32 | 7.54 | BDL* | | 475.01 | | 79.07 | | | 2.37 | |
| Black or African American | 24 | 39.97 | 14.05 | 0.91 | | 192.77 | | 55.47 | | | 1.39 | |
| Asian | 6 | 5.45 | 4.86 | BDL* | | 12.92 | | 4.41 | | | 0.81 | |
| Mix Race | 12 | 26.39 | 8.45 | BDL* | | 115.41 | | 42.16 | | | 1.60 | |
| Other | 3 | 5.94 | 6.00 | 4.29 | | 7.54 | | 1.62 | | | 0.27 | |
| **Ethnicity** | | | | | | | | | | | |  |
| Non-Hispanic | 53 | 31.45 | 6.00 | BDL* | | 475.01 | | 85.44 | | | 2.72 | |
| Hispanic/Latino | 35 | 31.29 | 11.84 | BDL* | | 209.25 | | 46.62 | | | 1.49 | |
| **Overall** | 88 | 31.35 | 7.54 | BDL* | | 475.01 | | 64.44 | | | 2.06 | |
| *BDL corresponds to values under 0.4 μg/cm²  **Since the study focuses on the accumulation of dust after a set period, the *N* only considers children whose net dust loading was greater than 0 μg/cm^2^ (88 out of 101), which emphasizes uptake of during play activity (mass of particles in post rinse was greater than pre rinse). As for the *N* in gender, parents did not provide information (1 out of 88)  ***No significant difference was obtained in any of the categories evaluated. | | | | | | | | | | | | |

| **Supplemental Table S6.** Net Dust Loading from Hand Rinses (Post minus pre-hand rinse) normalized by total hand area. | | | | | | | | | | | |  |
| --- | --- | --- | --- | --- | --- | --- | --- | --- | --- | --- | --- | --- |
|  | *N*** | Mass per Total Hands Surface Area (μg/cm^2^) | | | | | | Std. Dev. | | | Coeff. Var. | |
|  |  | Mean | Median | | Min | | Max | |  |  | | |
| **Region** | | | | | | | | | | | |  |
| North Carolina | 28 | 12.35 | 4.20 | BDL* | | 59.74 | | 16.17 | | | 1.31 | |
| Florida | 30 | 3.33 | 1.93 | BDL* | | 15.89 | | 3.65 | | | 1.10 | |
| Arizona | 30 | 17.80 | 4.08 | BDL* | | 167.64 | | 34.21 | | | 1.92 | |
| **Age** | | | | | | | | | | | |  |
| $6 \leq age<12$months | 15 | 21.77 | 6.98 | BDL* | | 167.64 | | 43.37 | | | 1.99 | |
| $1 \leq age<2$years | 23 | 8.60 | 1.99 | BDL* | | 71.80 | | 16.33 | | | 1.90 | |
| $2 \leq age<3$years | 23 | 7.67 | 2.12 | BDL* | | 37.39 | | 11.11 | | | 1.45 | |
| $3 \leq age<6$years | 27 | 10.32 | 2.76 | BDL* | | 69.11 | | 17.02 | | | 1.65 | |
| **Gender** | | | | | | | | | | | |  |
| Female | 45 | 10.66 | 2.73 | BDL* | | 71.80 | | 18.05 | | | 1.69 | |
| Male | 42 | 11.76 | 2.67 | BDL* | | 167.64 | | 27.14 | | | 2.31 | |
| **Race** | | | | | | | | | | | |  |
| White | 43 | 11.73 | 2.71 | BDL* | | 167.64 | | 27.77 | | | 2.37 | |
| Black or African American | 24 | 14.56 | 4.72 | BDL* | | 69.11 | | 19.67 | | | 1.35 | |
| Asian | 6 | 1.98 | 1.70 | BDL* | | 4.69 | | 1.62 | | | 0.82 | |
| Mix Race | 12 | 8.97 | 3.01 | BDL* | | 39.07 | | 14.03 | | | 1.57 | |
| Other | 3 | 2.06 | 1.99 | 1.56 | | 2.63 | | 0.54 | | | 0.26 | |
| **Ethnicity** | | | | | | | | | | | |  |
| Non-Hispanic | 53 | 11.12 | 4.19 | BDL* | | 71.80 | | 16.15 | | | 1.45 | |
| Hispanic/Latino | 35 | 11.14 | 2.12 | BDL* | | 167.64 | | 30.20 | | | 2.71 | |
| **Overall** | 88 | 11.13 | 2.72 | BDL* | | 167.64 | | 22.64 | | | 2.03 | |
| *BDL corresponds to values under 0.4 μg/cm²  **Since the study focuses on the accumulation of dust after a set period, the *N* only considers children whose net dust loading was greater than 0 μg/cm^2^ (88 out of 101). As for the *N* in gender, parents did not provide information (1 out of 88) | | | | | | | | | | | | |

| **Supplementary Table S7.** Estimated mass values (μg) for the final hand rinses of all children. If after the blank was applied the mass estimate was negative, the value was round up to zero. Additionally, those highlighted in pink used the average density obtained per region, where NC = North Carolina; FL = Florida; and AZ = Arizona. | | | | | | | | | | | | |
| --- | --- | --- | --- | --- | --- | --- | --- | --- | --- | --- | --- | --- |
| **NID** | **STATE** | **AGE CATEGORY** | **HISPANIC  OR LATIN** | **RACE** | **GENRE** | **PALMS SA (cm^2^)** | **TOTAL_HANDS SA (cm^2^)** | **DENSITY**  **g/cm^3^** | **MAX_MASS_ug** | **MIN_MASS_ug** | **AVG_MASS_ug** | **MEDIAN_MASS_ug** |
| 1 | NC | 3 | N | A | M | 134.65 | 383.39 | 1.70 | 1931.3 | 0.0 | 466.3 | 176.9 |
| 2 | NC | 2 | N | B | F | 103.61 | 298.90 | 1.52 | 706.0 | 386.2 | 555.9 | 559.0 |
| 3 | NC | 2 | N | B | F | 96.86 | 290.22 | 1.93 | 1599.3 | 863.1 | 1217.1 | 1185.8 |
| 4 | NC | 3 | N | B | M | 122.21 | 375.05 | 1.75 | 718.6 | 305.3 | 516.8 | 503.2 |
| 5 | NC | 4 | N | B | F | 127.85 | 349.13 | 1.61 | 4755.8 | 2266.5 | 3365.0 | 3165.9 |
| 6 | NC | 5 | R | R | F | 205.91 | 606.38 | 1.71 | 1460.9 | 833.7 | 1169.0 | 1223.4 |
| 7 | NC | 2 | N | B | M | 106.35 | 310.28 | 1.29 | 871.3 | 220.0 | 663.2 | 788.8 |
| 8 | NC | 2 | N | MIX | M | 107.11 | 302.34 | 1.81 | 3660.0 | 2936.5 | 3352.5 | 3461.1 |
| 9 | NC | 4 | N | MIX | M | 181.27 | 539.49 | 1.73 | 2503.5 | 1333.7 | 1686.9 | 1527.3 |
| 10 | NC | 4 | N | B | M | 143.90 | 426.84 | 1.43 | 2664.2 | 1603.2 | 2245.6 | 2205.4 |
| 11 | NC | 4 | N | B | M | 176.47 | 517.20 | 1.41 | 41.8 | 0.0 | 0.0 | 8.1 |
| 12 | NC | 2 | N | W | M | 107.58 | 305.19 | 1.60 | 1460.1 | 900.7 | 1128.0 | 1056.2 |
| 13 | NC | 1 | N | B | F | 67.48 | 198.58 | 1.61 | 1100.5 | 239.7 | 714.5 | 766.5 |
| 14 | NC | 4 | N | B | M | 128.52 | 373.99 | 1.61 | 5462.4 | 3294.3 | 4351.1 | 4241.9 |
| 15 | NC | 3 | N | MIX | F | 148.27 | 451.57 | 1.50 | 50661.8 | 4819.0 | 17481.7 | 7788.2 |
| 16 | NC | 2 | Y | W | M | 102.32 | 304.09 | 2.51 | 11007.5 | 7296.8 | 8685.8 | 8851.3 |
| 17 | NC | 4 | Y | W | F | 126.64 | 356.34 | 1.72 | 1610.9 | 623.7 | 867.9 | 646.0 |
| 18 | NC | 4 | N | B | M | 204.20 | 606.08 | 1.59 | 44546.5 | 14387.6 | 28870.2 | 29268.1 |
| 19 | NC | 4 | N | B | M | 155.27 | 429.50 | 1.63 | 2072.2 | 1053.0 | 1676.2 | 1860.5 |
| 20 | NC | 4 | N | B | M | 149.56 | 434.83 | 1.22 | 1057.7 | 563.6 | 901.3 | 960.1 |
| 21 | NC | 3 | N | MIX | F | 131.50 | 386.39 | 2.01 | 484.2 | 69.2 | 377.4 | 429.8 |
| 22 | NC | 1 | N | B | F | 88.42 | 261.78 | 1.36 | 19102.3 | 10590.6 | 16015.3 | 17473.3 |
| 23 | NC | 3 | N | B | F | 129.84 | 375.25 | 2.05 | 13311.8 | 9423.5 | 11007.4 | 10467.2 |
| 24 | NC | 4 | N | B | M | 176.07 | 515.01 | 2.07 | 19002.2 | 5603.3 | 12503.6 | 13514.2 |
| 25 | NC | 3 | N | B | M | 126.00 | 360.61 | 1.34 | 3203.3 | 212.8 | 1084.1 | 601.5 |
| **NID** | **STATE** | **AGE CATEGORY** | **HISPANIC  OR LATIN** | **RACE** | **GENRE** | **PALMS SA (cm^2^)** | **TOTAL_HANDS SA (cm^2^)** | **DENSITY**  **g/cm^3^** | **MAX_MASS_ug** | **MIN_MASS_ug** | **AVG_MASS_ug** | **MEDIAN_MASS_ug** |
| 26 | NC | 3 | N | B | M | 122.78 | 353.59 | 1.34 | 3689.5 | 2900.6 | 3507.7 | 3689.5 |
| 27 | NC | 1 | N | MIX | M | 76.33 | 219.36 | 1.20 | 141.3 | 0.0 | 16.9 | 8.0 |
| 28 | NC | 1 | N | MIX | F | 86.66 | 243.88 | 2.38 | 1638.4 | 733.1 | 1025.8 | 987.3 |
| 29 | NC | 2 | N | B | M | 105.42 | 305.55 | 1.61 | 9140.2 | 6297.6 | 8057.7 | 8353.2 |
| 30 | NC | 2 | Y | W | F | 84.47 | 233.57 | 1.61 | 305.6 | 22.9 | 122.2 | 43.8 |
| 31 | NC | 2 | Y | W | M | 87.57 | 251.81 | 1.61 | not collected | | | |
| 32 | NC | 3 | N | W | M | 114.94 | 329.04 | 1.48 | 1187.0 | 61.5 | 536.3 | 447.5 |
| 33 | NC | 2 | N | B | F | 83.61 | 245.86 | 1.60 | 392.8 | 261.4 | 306.3 | 287.0 |
| 34 | NC | 1 | N | B | M | 84.18 | 118.81 | 1.09 | 6025.9 | 2931.0 | 4064.7 | 3593.4 |
| 35 | NC | 1 | N | MIX | F | 68.28 | 189.67 | 1.28 | 171.9 | 45.3 | 93.9 | 87.5 |
| 36 | AZ | 3 | N | W | M | 97.11 | 269.46 | 1.53 | 6693.2 | 3719.2 | 5436.2 | 5528.1 |
| 37 | AZ | 4 | N | W | F | 144.00 | 404.57 | 1.53 | 4163.8 | 2845.4 | 3645.6 | 3857.2 |
| 38 | AZ | 4 | N | A | M | 156.38 | 446.28 | 1.40 | 1265.9 | 649.1 | 1021.5 | 1039.4 |
| 39 | AZ | 2 | N | W | F | 90.02 | 262.36 | 1.85 | 25655.8 | 9308.8 | 19729.6 | 21781.3 |
| 40 | AZ | 2 | N | W | F | 77.60 | 218.29 | 1.84 | 8856.8 | 3643.5 | 6474.1 | 6617.3 |
| 41 | AZ | 3 | N | MIX | F | 84.11 | 238.77 | 1.61 | 278.0 | 42.0 | 178.5 | 178.5 |
| 42 | AZ | 4 | Y | W | M | 108.37 | 297.58 | 1.12 | 1778.3 | 1108.7 | 1302.9 | 1198.0 |
| 43 | AZ | 2 | N | W | F | 83.98 | 229.42 | 1.75 | 1239.8 | 691.5 | 988.3 | 1124.5 |
| 44 | AZ | 4 | N | MIX | M | 154.53 | 456.49 | 1.54 | 28640.2 | 16775.3 | 24880.4 | 26791.1 |
| 45 | AZ | 4 | N | W | F | 141.26 | 409.80 | 1.88 | 19411.7 | 9336.0 | 14078.2 | 13573.5 |
| 46 | AZ | 2 | N | W | F | 74.95 | 207.35 | 1.51 | 858.1 | 451.6 | 679.9 | 654.9 |
| 47 | AZ | 3 | N | W | F | 87.09 | 231.40 | 1.60 | 1091.6 | 762.6 | 985.3 | 1067.5 |
| 48 | AZ | 4 | Y | W | M | 131.04 | 371.70 | 1.46 | 317.8 | 24.1 | 120.6 | 84.0 |
| 49 | AZ | 2 | N | W | M | 93.10 | 258.59 | 1.74 | 877.2 | 606.4 | 754.0 | 767.9 |
| 50 | AZ | 3 | N | B | F | 104.44 | 292.40 | 1.60 | 785.3 | 331.8 | 634.3 | 663.5 |
| 51 | AZ | 3 | N | W | F | 89.39 | 246.73 | 1.56 | 1701.2 | 955.5 | 1477.0 | 1607.4 |
| 52 | AZ | 4 | Y | B | F | 117.79 | 328.57 | 1.53 | 33091.5 | 20367.6 | 25457.1 | 25273.2 |
| 53 | AZ | 4 | Y | B | F | 140.52 | 376.47 | 1.93 | 2660.1 | 997.8 | 1942.0 | 1927.3 |
| 54 | AZ | 3 | N | W | M | 116.39 | 316.37 | 1.91 | 3947.0 | 2477.7 | 3481.4 | 3527.2 |
| **NID** | **STATE** | **AGE CATEGORY** | **HISPANIC  OR LATIN** | **RACE** | **GENRE** | **PALMS SA (cm^2^)** | **TOTAL_HANDS SA (cm^2^)** | **DENSITY**  **g/cm^3^** | **MAX_MASS_ug** | **MIN_MASS_ug** | **AVG_MASS_ug** | **MEDIAN_MASS_ug** |
| 55 | AZ | 2 | N | MIX | F | 89.64 | 251.47 | 1.35 | 809.5 | 171.4 | 545.9 | 539.1 |
| 56 | AZ | 1 | Y | W | F | 59.93 | 159.49 | 1.37 | 1358.6 | 1175.1 | 1261.7 | 1249.1 |
| 57 | AZ | 3 | Y | W | M | 106.16 | 288.94 | 1.54 | 1280.4 | 372.9 | 706.1 | 562.1 |
| 58 | AZ | 3 | Y | W | F | 93.17 | 257.74 | 1.33 | 15604.2 | 3736.9 | 8372.4 | 6707.1 |
| 59 | AZ | 1 | Y | W | M | 83.57 | 236.80 | 1.50 | 90253.8 | 21040.7 | 39848.6 | 29316.2 |
| 60 | AZ | 1 | Y | W | M | 63.81 | 168.34 | 1.52 | 41.2 | 0.0 | 0.0 | 0.0 |
| 61 | AZ | 3 | Y | W | M | 123.59 | 356.22 | 1.86 | 999.4 | 640.9 | 898.7 | 947.4 |
| 62 | AZ | 2 | N | W | O | 68.21 | 193.08 | 1.46 | 1605.9 | 835.3 | 1140.0 | 1067.3 |
| 63 | AZ | 2 | N | W | F | 74.06 | 198.74 | 1.14 | 372.1 | 0.0 | 75.8 | 0.0 |
| 64 | AZ | 2 | N | W | F | 78.87 | 216.60 | 1.44 | 422.7 | 318.8 | 364.1 | 357.8 |
| 65 | AZ | 3 | N | W | M | 118.71 | 323.49 | 1.60 | 2564.8 | 905.1 | 1790.1 | 1571.6 |
| 66 | AZ | 2 | Y | W | F | 81.77 | 224.41 | 1.22 | 90.8 | 0.0 | 16.0 | 0.0 |
| 67 | AZ | 2 | Y | W | M | 74.69 | 211.60 | 1.74 | 1000.0 | 294.2 | 686.6 | 632.3 |
| 68 | AZ | 3 | N | W | F | 106.67 | 307.16 | 1.13 | 1569.7 | 458.5 | 906.6 | 685.1 |
| 69 | FL | 4 | Y | W | M | 136.14 | 375.72 | 1.60 | 128.0 | 41.5 | 93.4 | 108.8 |
| 70 | FL | 3 | Y | W | M | 108.49 | 318.90 | 1.59 | 487.4 | 130.9 | 222.3 | 159.6 |
| 71 | FL | 3 | N | B | F | 109.00 | 302.78 | 1.42 | 1427.6 | 593.9 | 838.0 | 720.1 |
| 72 | FL | 1 | N | B | F | 76.45 | 217.67 | 1.86 | 4147.0 | 1140.5 | 1968.7 | 1522.4 |
| 73 | FL | 4 | Y | W | M | 151.08 | 419.99 | 2.11 | 1180.4 | 899.7 | 1051.2 | 1072.7 |
| 74 | FL | 4 | Y | W | M | 117.05 | 321.90 | 1.79 | 1294.5 | 323.4 | 885.0 | 875.0 |
| 75 | FL | 4 | Y | W | F | 143.91 | 405.40 | 1.16 | 800.6 | 421.2 | 586.4 | 587.8 |
| 76 | FL | 4 | N | W | F | 128.73 | 371.06 | 1.87 | not collected | | | |
| 77 | FL | 3 | Y | W | F | 95.33 | 257.90 | 1.73 | 5747.3 | 1465.5 | 2964.1 | 2449.7 |
| 78 | FL | 2 | Y | O | F | 88.54 | 266.87 | 1.55 | 687.6 | 302.1 | 555.4 | 591.6 |
| 79 | FL | 3 | Y | W | F | 98.56 | 277.39 | 1.83 | 698.4 | 371.9 | 543.2 | 551.7 |
| 80 | FL | 3 | Y | W | M | 95.81 | 277.96 | 1.10 | 1499.7 | 0.0 | 311.6 | 40.2 |
| 81 | FL | 2 | Y | MIX | F | 105.16 | 290.05 | 2.42 | 615.5 | 199.9 | 369.5 | 352.1 |
| 82 | FL | 4 | N | W | F | 129.55 | 366.00 | 1.70 | 2474.4 | 1166.9 | 1839.3 | 1676.3 |
| 83 | FL | 4 | N | MIX | M | 146.56 | 409.78 | 1.19 | 0.0 | 0.0 | 0.0 | 0.0 |
| **NID** | **STATE** | **AGE CATEGORY** | **HISPANIC  OR LATIN** | **RACE** | **GENRE** | **PALMS SA (cm^2^)** | **TOTAL_HANDS SA (cm^2^)** | **DENSITY**  **g/cm^3^** | **MAX_MASS_ug** | **MIN_MASS_ug** | **AVG_MASS_ug** | **MEDIAN_MASS_ug** |
| 84 | FL | 4 | Y | B | M | 111.15 | 299.73 | 0.76 | 384.7 | 97.4 | 242.3 | 279.5 |
| 85 | FL | 4 | Y | W | M | 210.75 | 606.33 | 1.61 | 1128.7 | 1012.8 | 1050.8 | 1035.3 |
| 86 | FL | 4 | Y | O | M | 121.52 | 334.11 | 0.98 | 744.9 | 418.1 | 521.8 | 479.0 |
| 87 | FL | 4 | Y | MIX | F | 130.71 | 368.45 | 1.91 | 2746.1 | 1239.9 | 2021.1 | 2116.2 |
| 88 | FL | 2 | N | A | F | 82.88 | 238.05 | 1.63 | 810.4 | 0.0 | 399.1 | 569.0 |
| 89 | FL | 2 | Y | W | M | 91.11 | 260.32 | 1.61 | 1949.3 | 1547.0 | 1823.7 | 1868.8 |
| 90 | FL | 4 | N | A | F | 141.72 | 390.66 | 1.93 | 2603.9 | 1191.5 | 1941.3 | 2005.8 |
| 91 | FL | 3 | Y | W | M | 109.45 | 310.34 | 1.05 | 945.2 | 416.7 | 667.9 | 656.4 |
| 92 | FL | 4 | Y | MIX | F | 140.39 | 393.91 | 1.41 | 165.7 | 86.6 | 129.3 | 134.6 |
| 93 | FL | 4 | N | A | M | 130.96 | 354.50 | 1.61 | 987.1 | 31.3 | 231.8 | 50.6 |
| 94 | FL | 4 | N | A | M | 126.65 | 322.24 | 1.54 | 1245.7 | 887.5 | 1071.2 | 1123.7 |
| 95 | FL | 1 | Y | W | F | 76.65 | 212.67 | 1.61 | 2914.7 | 673.3 | 1536.4 | 1360.4 |
| 96 | FL | 1 | N | W | F | 72.00 | 204.92 | 1.63 | 1192.3 | 525.9 | 927.7 | 990.2 |
| 97 | FL | 1 | Y | MIX | M | 87.90 | 244.89 | 1.65 | 3114.6 | 2354.3 | 2922.9 | 3081.6 |
| 98 | FL | 1 | Y | O | M | 94.39 | 270.26 | 1.66 | 1439.2 | 502.8 | 921.8 | 932.8 |
| 99 | FL | 1 | Y | W | F | 78.91 | 226.03 | 1.38 | 638.4 | 438.6 | 504.2 | 496.5 |
| 100 | FL | 1 | Y | W | M | 75.32 | 205.51 | 1.76 | 402.2 | 65.3 | 204.6 | 195.8 |
| 101 | FL | 1 | Y | W | M | 71.15 | 206.40 | 2.22 | 4917.6 | 1802.2 | 3280.6 | 3116.4 |
